# Mechanistic Investigations of a Novel Lactam Antifungal Agent Against *Candida albicans*

**DOI:** 10.1111/apm.70258

**Published:** 2026-09-03

**Authors:** Hafsa Abduljalil, William Johnston, Mark Butcher, Neil Parry, Joanne O'Keeffe, Yvonne Davies, Gordon Ramage

**Affiliations:** ^1^ Research Centre for Health, School of Health and Life Sciences Glasgow Caledonian University Glasgow UK; ^2^ Oral Sciences Research Group, Glasgow Dental School, School of Medicine, Dentistry and Nursing College of Medical, Veterinary and Life Sciences Glasgow UK; ^3^ Unilever R&D Wirral UK; ^4^ Penrhos Bio Limited London UK

**Keywords:** biofilm, *Candida*, hyphae, lactam, vacuole

## Abstract

*Candida albicans* presents a major, yet underestimated challenge in clinical settings due to its increased resistance to treatment. This study intended to focus on the utility of a novel proprietary lactam molecule, a quorum sensing (QS) inhibitor, against *C. albicans* . We assessed isolate susceptibility to lactam treatment. In addition, RNA‐Seq was used to assess lactam‐induced transcriptional changes in phenotypically distinct *C. albicans* strains. The mechanistic basis of these transcriptional responses and a series of biochemical assays were performed using the reference strain SC5314. Our findings show that lactam influences multiple cellular processes, including cell wall integrity, efflux pump activity, oxidative stress responses and biofilm formation, with effects differing markedly between high and low biofilm formers. Vacuolation was observed with prolonged exposure to the lactam, and related genes in this process were identified and the phenotype confirmed. These results, supported by pathway analyses and mechanistic validation experiments, indicate that lactam's antifungal activity does not rely on a single mode of action. Rather, it appears to involve multiple mechanisms that vary across heterogeneous *C. albicans* isolates. These data indicate the potential therapeutic benefit of a novel fungicidal antifungal agent with broad‐spectrum activity.

## 1 Introduction

Fungi are increasingly acknowledged as significant human pathogens. It is estimated that invasive fungal infections affect around 6.5 million people globally, resulting in over 2.5 million deaths each year [1]. In 2022, the World Health Organisation (WHO) published its priority fungal pathogens list [2], and among the critical priority pathogens is *C. albicans* which is considered the paradigm biofilm‐forming fungal pathogen [1]. Its capacity to alter its morphology and undergo the transition from yeast to the hyphal form is a property important for its pathogenesis, but also for biofilm development [3]. Critically, biofilms represent an important issue for clinical management because they exhibit elevated tolerance to antifungal therapy that is independent of defined genetic resistance mechanisms [4, 5]. Potent anti‐biofilm agents are limited to liposomal amphotericin B and the echinocandins [6], but their effectiveness is limited by resistance, inadequate penetration into sequestered infection sites, and drug‐specific side effects that necessitate regular patient monitoring and reassessment [7].

Discovery of new antifungal effectors that exhibit anti‐biofilm properties would be advantageous for improving patient care. Quorum sensing molecules (QSMs) that regulate microbial biofilm behaviour are emerging as promising tools in combating infections and antimicrobial resistance. Our group has recently reported that the QSM lactams are highly effective against a variety of fungi, exhibiting the ability to inhibit and kill fungal cells in both simple and complex biofilm model systems, and influence the biofilm composition [8]. Lactams (dihydropyrrol‐2‐one's [DHPs]) are structural analogues of fimbrolides derived from the red alga *Delisea pulchra* [9], have been shown to inhibit acyl homoserine lactone (AHL)‐mediated quorum sensing in Gram‐negative bacteria, a critical mechanism for biofilm formation [10]. Despite our mechanistic insights into how these molecules influence biological behaviours of bacteria, we have little understanding of how lactams influence fungi beyond their antimicrobial properties. This in vitro study therefore aimed to investigate the phenotypic and transcriptomic responses of *C. albicans* to lactam treatment. In this study, we demonstrate for the first time that lactams enter cells and gradually toxify the hyphal elements leading to vacuolar overload and cell death, and despite cell defence mechanisms being activated, cells cannot be resuscitated.

## 2 Materials and Methods

### 2.1 Microbial Growth Conditions and Standardisation

Yeast cultures were maintained on Sabouraud agar at 30°C, as previously described and stored at 4°C until required [8]. The *C. albicans* clinical isolates used were obtained from denture stomatitis patients at Glasgow Dental Hospital (BC isolates), as previously described [11, 12]. Briefly, cultures of *C. albicans* SC5314 type strain, high biofilm former (HBF [BC146]) and low biofilm former (LBF [BC023]) clinical isolates were prepared overnight in yeast peptone dextrose broth (YPD, Sigma‐Aldrich, UK) and incubated for 18 h at 30°C. These were then pelleted by centrifugation for 5 min and washed twice with Phosphate Buffered Saline (PBS). Finally, yeast cells were counted using a Neubauer haemocytometer.

### 2.2 Investigating the Killing and Inhibitory Effects of Lactam

To assess kill kinetics, *C. albicans* SC5314 was prepared as biofilms using standard methods in 96‐well plates for 24 h. Cells were standardised 1 × 10^6^ cells/mL in Roswell Park Memorial Institute (RPMI)‐1640 (Sigma‐Aldrich) medium and grown for 24 h. Biofilms were then treated with lactam 488 (Unilever R&D, Wirral, UK) at 7 (pMIC), 14 (2× pMIC) and 28 μg/mL (4× pMIC) for 0, 2, 4, 6, 8 and 24 h. Planktonic minimum inhibitory concentration (pMIC) refers to the lowest concentration of lactam at which there was no visual growth of the fungal cells compared with the drug‐free control. Biofilm kill was quantified using the metabolic XTT assay (2,3‐bis(2‐methoxy‐4‐nitro‐5‐sulfophenyl)‐2H‐tetrazolium‐5‐carboxanilide [Fisher Scientific, Paisley, UK]) [13], and metabolic activity was measured at 492 nm on an Omega plate reader and the resultant reduction in absorbance was compared to positive controls. Next, to assess inhibition, the growth kinetics of each isolate (SC5314, BC146, BC023) was assessed in the presence of three concentrations (1.75, 3.5 and 7 μg/mL) of lactam 488 (Unilever R&D, Wirral, UK). Cells were assayed using the M27‐A3 CLSI standardised yeast broth microdilution assay format [14]. Briefly, cells were standardised to 2 × 10^4^ cells/mL in RPMI‐1640 medium and added to a round bottomed 96‐well microtiter plate (Corning Incorporated, Corning, NY, USA) containing the three concentrations of lactam. The plate was incubated at 37°C for 24 h in an Omega plate reader (BMG Labtech) with absorbance measured at 570 nm every 15 min. Untreated control of *C. albicans* cells was also included. Light microscopy was then used to examine inhibition of early *C. albicans* biofilms treated with lactam. SC5314 cells were standardised 1 × 10^6^ cells/mL in RPMI‐1640 grown for 4 h in a 24‐well plate at 37°C. Biofilms were gently washed with PBS before being treated with 7 μg/mL of lactam or with media control. Light microscopy was also performed at 1, 4, and 24 h post treatment using the EVOS FL Cell Imaging System (ThermoFisher Scientific, Waltham, MA, USA). Experiments were repeated at three independent occasions with four technical repeats.

### 2.3 Assessing the Transcriptional Response to Lactam Treatment

Transcriptional analysis of *C. albicans* in response to lactam was performed using RNA sequencing methodology, as previously described [15, 16]. Due to the high cost of RNA‐seq, only 2 strains (1 HBF, and 1 LBF) of *C. albicans* were selected for transcriptome study. Overnight cultures of *C. albicans* SC5314 and the LBF clinical isolate (BC023) were standardised at cellular density of 1 × 10^7^ cells/mL in RPMI medium containing lactams (3.5 and 1.75 μg/mL). Untreated *Candida* cells were also included as positive controls. Cells were incubated in a shaking incubator at 37°C for 4 h. After incubation the cultures were centrifugated at 3000 rpm for 20 min at 4°C. The pellets were washed and resuspended in PBS and RNA then extracted using RiboPure RNA purification kit for yeast (Thermo Scientific, Loughborough, UK). The quantity and quality of extracted RNA were evaluated using Bioanalyzer system (Agilent, USA). RNA samples were sequenced using Illumina NextSeq2000 sequencing platform at the Glasgow University Polyomics facility. This experiment was performed at three independent occasions.

The raw fastq reads were first uploaded to University of Glasgow Galaxy server (http://antioch.tcrc.gla.ac.uk) for processing, before being analysed by R software. Galaxy server contains all the tools for necessary data processing. Quality check of the raw data was performed by implementing FastQC, and Trimmomatic software was applied to remove adaptors, and low‐quality reads of < 30 bases [17]. Reference genome and gene model annotation files were downloaded from (http://www.candidagenome.org). Hisat2 was then used to map the trimmed high‐quality reads to *C. albicans* SC5314 genome (*Candida* Genome database). Reads that were mapped to a feature on *C. albicans* genome were counted with the use of the tool HTSeq‐count and imported to R for further analysis. Differential expression analysis was primarily conducted using the DESeq2 R package. DESeq2 applies a negative binomial model to estimate gene abundance and assess differential expression between experimental conditions. The ClueGO application in Cytoscape (Version: 3.8.0) software was used to generate gene ontology networks.

### 2.4 Transcriptomic Expression Validation in Lactam‐Treated Cells

To assess *C. albicans* biofilms exhibiting phenotypic changes, the expression of some key genes of interest was examined. Biofilms of *C. albicans* SC5314 were grown for 4 h at 37°C and subsequently treated with 7 μg/mL lactam for an additional 4 h. After incubation, biofilms were removed by scraping, and RNA extracted using TRIzol extraction, as per the manufacturer's protocol (Thermo Fisher Scientific, Paisley, UK). Extracted RNA was converted to cDNA with a high‐capacity cDNA reverse transcription kit (Thermo Scientific, Loughborough, UK). qPCR was carried out using the Step‐One plus real time PCR machine (Life Technologies). The following profile was used: 50°C for 2 min, 95°C for 2 min, followed by 40 cycles of 95°C for 3 s and 60°C for 30 s. The list of primers for genes assessed is in Table 1. Gene expression was analysed for *C. albicans* using the ΔΔCT method, after normalising CT values to *ACT1* housekeeping gene [18]. No‐reverse transcription samples were included.

**TABLE 1:** *Candida albicans* primers used for quantitative and real time PCR.

Primer name	Sequence (5¢‐3¢)	Function
*ACT1*	F—AAGAATTGATTTGGCTGGTAGAGA	Housekeeping
	R—TGGCAGAAGATTGAGAAGAAGTTT	
*HWP1*	F‐ GCTCAACTTATTGCTATCGCTTATTACA	Hyphal wall protein
	R—GACCGTCTACCTGTGGGACAGT	
*SOD5*	F—ACGAGGGACACGGCAATGCT	Oxidative stress response
	R—GCGCCATTACCTTGAGGAGCAGTA	
*CDR2*	F—GCTGGTATCATTGCTGATAT	Efflux pump
	R—TTCGATGGATTGTTGAACAC	
*ERG2*	F—TCCTGGTGCATTGATTCCCG	Ergosterol biosynthesis
	R—ATGATTCACCGGGCATAGCA	
*ERG11*	F—TTTAGTTTCTCCAGGTTATGCTCAT	Ergosterol biosynthesis
	R—ATTAGCTTTGGCAGCAGCAGTA	
*UPC2*	F—TCCATCCTTGACCCCTAGTCCT	Ergosterol biosynthesis
	R—CGGCTGAGTTTTGATGTCTTGA	
*PMC1*	F—ACGCCGTTATTACCGCTGTG	Vacuole calcium pump
	R—CAATGGCAGCAAGGAACCCA	
*YVC1*	F—ATGTGCCTCTCCGTTAATGTGGTC	Vacuole calcium pump
	R—TGCGTGGCTTCGTTCTTACCATC	
*VMA4*	F—ATGTTGCCAAAGAAGCCATAAC	Vacuole ATPase
	R—ACCACCAGCGATATCTTTAGC	
*VCX1*	F—GTGCCGATGCGATCTTGAATGAAC	Vacuole calcium pump
	R—ACAATACGACCAAGCCAGCAGTC	
*PMR1*	F—ACTGCTAGAGACACGACCATGACC R—TGGAATAATGACGGCACGACAAGG	Golgi Calcium pump

### 2.5 Investigating the Effect of Lactam on Cell Membrane Integrity

Ergosterol powder (Sigma‐Aldrich, Dorset, UK) was dissolved in Tween 80 and ethanol at a ratio of 1∶1, to prepare a 5 mM stock solution. Utilising the M27‐A3 CLSI standardised broth microdilution method, dilutions of lactam (0.8 to 28 μg/mL) were added simultaneously to *C. albicans* SC5314 cells in RPMI medium and dispensed in a 96‐well flat‐bottomed plate in the ± ergosterol (50 μM), as previously studied [19]. After 4 h incubation at 37°C, media were discarded, biofilms were gently washed with PBS, and XTT assay was performed to assess viability.

### 2.6 Investigating the Effect of Lactam Induced Efflux Pump Activity

Efflux pump activity of lactam‐treated *C. albicans* SC5314 biofilms was assessed using an alanine β‐naphthylamine [MC‐005,556 (Ala‐Nap)] fluorescent assay, as previously described [20]. In this assay, Ala‐Nap is enzymatically cleaved inside the cells to produce highly fluorescent β‐naphthylamine. Higher levels of fluorescence reflect low efflux pump activity and vice versa. Biofilm cells of *C. albicans* SC5314 were grown directly as biofilms on black flat bottomed microtiter plates (Costar 3603; Corning, NY) at 37°C for 24 h and subsequently treated with subinhibitory concentrations of lactam (3.5 μg/mL and 1.75 μg/mL) for 2, 4 and 6 h. Following incubation, biofilms were washed with assay buffer (K2HPO4 [50 mM], MgSo4 [1 mM], and glucose [0.4%]) and the reaction was initiated by the addition of 100 μg/mL Ala‐Nap (Sigma‐Aldrich, Gillingham, United Kingdom). Fluorescence was quantified at 30s intervals for 1 h at 37°C using a fluorescence plate reader at an excitation and emission wavelengths 355/460 nm, respectively. Next, the efflux pump inhibitor (EPI; l‐Phe‐l‐Arg‐β‐naphthylamine dihydrochloride [MC‐207,110]; Sigma‐Aldrich, Gillingham, United Kingdom) was used in combination with lactam to determine if antifungal activity could be enhanced. First, to determine the effects of EPI on the antifungal activity of lactam, a checkerboard titration assay was performed as previously described (Abduljalil et al.). A single concentration of lactam that demonstrated synergy with EPI was selected for further testing. Similarly, a single concentration of EPI that exhibited no antifungal activity was used in subsequent experiments. Lactam (0.1 μg/mL) was tested against *C. albicans* SC5314 cells (24 h) and 24 h‐biofilms (4 h and 24 h treatment) ± (64 μg/mL) and incubated overnight at 37°C. Biofilms were then washed with PBS, before viability was calculated using the XTT assay as described above.

### 2.7 Investigating the Effect of Lactam Inhibition on Hyphal Morphogenesis

To assess the impact of lactam on filamentation, an assay was performed as previously described [21]. Briefly, *C. albicans* SC5314 were grown overnight in YPD medium at 30°C with shaking. One‐millilitre aliquots of overnight cultures were centrifuged, washed twice and resuspended with an equal volume of PBS. For solid filamentation phenotypic assays, 1 μL of resuspended cells were plated in a grid on non‐inducing (YPD, Sigma‐Aldrich, Dorset, UK) and inducing (Spider [Nutrient broth of 10 g, 10 g d‐mannitol, 2 g K_2_HPO_4_, 20 g agar]) agar plates ± lactam (3.5 μg/mL and 1.75 μg/mL). Non‐inducing plates were incubated at 30°C, and inducing plates were incubated at 37°C. Colonies and colony edges were imaged 4–5 days post‐incubation. Colony edges were imaged on an EVOS FL Cell Imaging System at 4× and 2× magnification. For liquid filamentation assays, 10 μL of washed cells were added to 2 mL of pre‐warmed Spider media in bijoux tubes using the same experimental parameters. Incubated cells were grown in a shaking incubator at 37°C for 3 h and imaged at 40× magnification on an EVOS FL Cell Imaging System. All conditions were tested in triplicate on three separate occasions.

### 2.8 Investigating the Effect of Lactam on Vacuole Function

RNA‐seq analysis identified the top upregulated gene (C2_02490C_A) in the lactam‐treated HBF group as being associated with vacuolar function (Table 1). This suggests that the phenotypic changes observed in early *C. albicans* biofilms treated with 7 μg/mL lactam may be attributable to vacuolar dysfunction and a consequent increase in membrane permeability following lactam exposure. Vacuole membrane permeability (VMP) was assessed in early biofilms utilising the ROS indicator 5‐(6)‐carboxy‐2′,7′‐dichlorofluorescein diacetate (C‐DCFDA, Biotium, California, USA). This fluorogenic compound can diffuse readily into the vacuole lumen, which becomes fluorescent and membrane impermeant upon hydrolysis of the acetate groups by esterases present inside the vacuole. Upon increase in VMP, this fluorescent compound can leak from the vacuole and diffuse easily into the cytosol of the fungal cell [22]. Lactam treated (7 μg/mL) and untreated biofilms were stained with 10 μM C‐DCFDA. For optimal hyphal visualisation, 5 μM calcofluor white (CFW) (Invitrogen, Paisley, UK) was used to stain the fungal cell wall. Biofilms were incubated in the dark for 20 min and excess stain was washed with sterile water. 2% paraformaldehyde (Sigma‐Aldrich, Gillingham, United Kingdom) was used for 1 h to fix the stained biofilms. Slides were maintained in the dark before imaging, and stained biofilms were visualised by confocal laser scanning microscopy (CLSM) (Upright Zeiss LSM 880). 504ex/529emnm, and 365ex/435emnm were used for excitation/emission wavelengths for visualising C‐DCFDA and CFW, respectively.

Next, the vacuole function was investigated indirectly by evaluating the role of calcium on the primary calcium (Ca^2+^) storage organelle [23]. *C. albicans* SC5314 cells were tested in the presence of lactam in a concentration range between 0.4 to 7 μg/mL ± calcium chloride (CaCl_2_ [10 mM]), using a standard broth microdilution assay as above described. The calcium chelator (Ethyleneglycol‐ *bis*(β‐aminoethyl)‐N,N,N′,N′‐tetraacetic Acid [EGTA]) (Sigma‐Aldrich, Dorset, UK) was also tested with similar concentration of lactam to examine if removing calcium from the media would improve the antifungal activity of lactam. *C. albicans* cells (SC5314) were then added to all plates and incubated for 24 h at 37°C. Following removal of media, XTT was then performed to assess viability. Fluorescent images were also obtained using 5 μM calcofluor, as described above. Experiments were repeated at three independent occasions with four technical repeats.

### 2.9 Investigating Lactam Induced Sensitisation During Combination Treatment

A checkerboard microdilution assay was performed to assess the biofilm inhibiting properties from combining lactam (0.02–28 μg/mL) with the following antimicrobials: polymyxin B (8–256 μg/mL), amphotericin B (0.015–0.5 μg/mL), chitosan (4.68–150 μg/mL), caspofungin (0.03–2 μg/mL), fluconazole (0.125–8 μg/mL), and H_2_O_2_ (0.015–0.48%). Checkerboard plates were prepared, incubated at 37°C for 24 h, and an XTT assay was performed to assess viability, as previously described [24]. To further verify testing findings, one sub‐inhibitory concentration of each drug combination that has shown a synergy (≥ 70% inhibition rate) was selected and tested for its biofilm inhibiting effect against cells of *C. albicans* SC5314.

### 2.10 Statistical Analysis

All graphs, data distribution and statistical analysis were performed using GraphPad Prism version 10 (GraphPad, San Diego, CA, USA). Before analysis, data distributions were assessed using a D'Agostino‐Pearson omnibus normality test, unless otherwise stated. Kruskal‐Wallis with Dunn's tests were used to determine the *p*‐value (p) for multiple comparisons when data were not normally distributed. Parametric data of multiple comparisons were analysed by analysis of variance (ANOVA) with Dunnett's tests to compare every mean to the control mean. Differences were considered statistically significant if *p* < 0.05.

## 3 Results

### 3.1 Lactam Kills and Inhibits *Candida albicans* Cells

**FIGURE 1:**
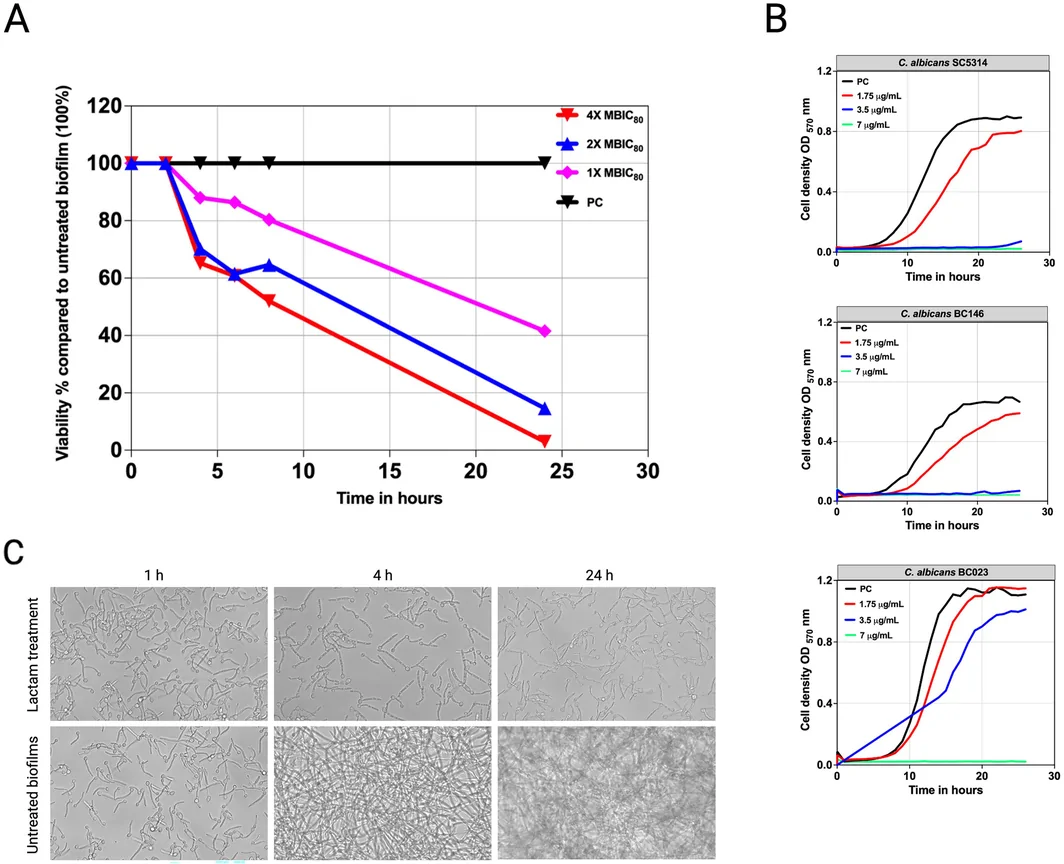
Lactam induced killing and inhibition of *Candida albicans* . (A) Assessment of kill kinetics of lactam against *C. albicans* biofilms. Twenty‐four hour‐biofilms of *C. albicans* (SC5314) treated with three concentrations of lactam (7 μg/mL [pMIC], 14 μg/mL [2X pMIC] and 28 μg/mL [4× pMIC]) Each bar represents the mean of data obtained from triplicates of three independent experiments. Error bars represent the standard error of the deviation. PC refers to positive untreated control. (B) Growth kinetics of *C. albicans* cells treated with lactam. Planktonic cells of *C. albicans* reference strain (SC5314) HBF (BC146) and LBF (BC023) clinical isolates were incubated aerobically with different concentrations of lactam (1.75, 3.5 and 7 μg/mL) at 37°C in a microtiter plate reader with absorbance readings (OD at 570 nm) were taken every 15 min for 26 h. (C) Light microscopic imaging of early biofilms of *C. albicans* SC5314 with lactam. *C. albicans* cells were grown as biofilms for 4 h and then treated with lactam (7 μg/mL) for 24 h and images were taken every 1, 4, 24 h. Scale bar represents 100 μm at ×40.

First, to confirm the antifungal properties of lactam, kill kinetics was performed against *C. albicans* SC5314 cells using lactam at 7, 14 and 28 μg/mL. Metabolic reduction was only observed in a concentration‐dependent effect after 8 h, with 14 and 28 μg/mL demonstrating an equivalent level of killing at 4 and 6 h (*p* < 0.0001), and no significant killing was observed between any of the concentrations at 2 h post treatment. At 8 h, killing was observed for 20%, 38% and 44%, respectively, and at 24 h a 60%, 83% and 98% (*p* < 0.0001) kill was observed, respectively (Figure 1A).

Next, lactam induced biofilm inhibition was evaluated at lower concentrations through assessing the growth kinetics of *C. albicans* SC5314 and two phenotypically distinct clinical isolates (Figure 1B). Data showed that a concentration of 1.75 μg/mL lactam was ineffective against all three *C. albicans* tested, whereas with 3.5 and 7 μg/mL there was complete inhibition of growth of both HBF strains (SC5314, BC146). Interestingly, the LBF showed a notable degree of tolerance when treated with 3.5 μg/mL of lactam. Growth kinetics were analysed by calculating the total area under the growth curve (AUC), which provides an integrated measure of cumulative biomass production over time. AUC was computed using the trapezoidal rule, where the area between each pair of consecutive time points was approximated as a trapezoid and summed to generate the total value for each condition. This approach allowed quantitative comparison of overall growth between treatments. Untreated *C. albicans* strains (SC5314, BC146, and BC023) exhibited the highest AUC values (12.88, 9.64, and 16.54, respectively), indicating robust and sustained growth. The 1.75 μg treatment produced moderate AUC values (9.060, 6.71, and 14.9), reflecting partial inhibition. In contrast, the 3.5 μg treatment resulted in markedly reduced AUC values for the two HBF strains (0.7970 and 1.35) and a moderate reduction for the LBF strain (9.95). The highest lactam concentration (7 μg/mL) produced the strongest inhibition across all three strains, yielding the lowest AUC values (0.4805, 1.07, and 0.58 for SC5314, BC146, and BC023, respectively).

Additionally, light microscopic examination of early 4 h *C. albicans* biofilms revealed distinct phenotypic change when incubated with 7 μg/mL lactam, which remained after 24 h of lactam exposure (Figure 1C). It was noted that distinct vacuole structures could be observed as oval to rectangular bands in *Candida* hyphae.

### 3.2 Transcriptional Response to Lactam Is Influence by Fungal Phenotype

**FIGURE 2:**
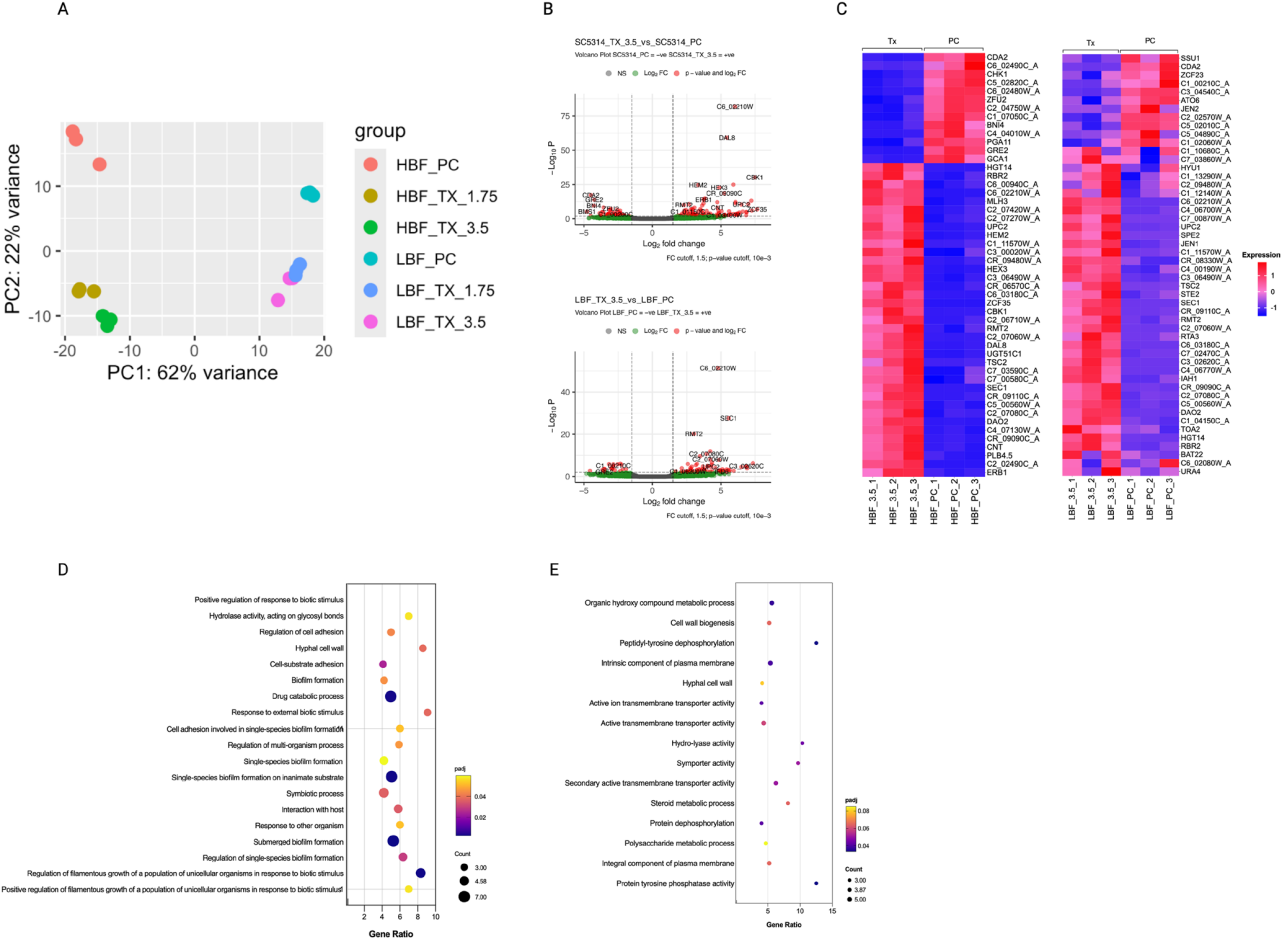
Overview of the transcriptional analysis of *C. albicans* strains treated with lactam. (A) The principal component analysis of data at all time points. (B) Volcano plot of the significantly differentially expressed genes of *C. albicans* reference strain, HBF, and LBF clinical isolates. Genes of interest can be identified where expression levels are above or below the Log_2_FC cut‐offs of −1.5 and 1.5 (green) and those that are also highly significant (*p* < 0.001; red). (C) Heatmap of the top 50 significantly differentially expressed genes of *C. albicans* reference strain, HBF, and LBF clinical isolates. Gene expression values were standardised using z‐scores, with red indicating upregulation, blue indicating downregulation. Each row represents one gene, and each column represents a single sample of the experimental groups; treated (Tx) and untreated (PC). Enriched biological processes within the downregulated genes in HBF samples treated with lactam (3.5 μg/mL). (D) Gene network shows the interaction between downregulated genes. Genes of similar function are grouped together to form nodes (circles) and nodes with similar functions are linked by edges lines. Nodes are coloured by levels of significance and node size increases. (E) Dotplot showing enriched GO terms related to downregulated genes within same group.

Given that lactam was shown to kill and inhibit *C. albicans* , combined with it appearing to induce vacuolation (a characteristic of cellular toxicity), we then aimed to understand its mode of action by undertaking transcriptomics on cells exposed for 4 h compared to untreated controls from two phenotypically distinct isolates. Although phenotypic changes were observed in early biofilms, planktonic *C. albicans* cells were selected for transcriptomic analysis to maximise RNA yield and for practical reasons. Multivariate analysis by Principal Component Analysis (PCA) was performed, where PC1 showed a variance of 62% between samples by the type of *Candida* strain (HBF and LBF). The second variance between samples (PC2) was 22% between treated and untreated samples (regardless of the type of the *Candida* strain) (Figure 2A). This indicated that *Candida* phenotype has a significant influence on the transcriptional response to lactam treatment, with samples clustering according to their phenotype and treatment concentration.

Differential gene expression (DGE) analysis revealed that the HBF treatment group treated with 3.5 μg/mL of lactam exhibited the highest number of significantly DGEs (*n* = 115) among all treatment groups, with a cut‐off of 1.5 for fold change and 10e‐3 for *p‐*adjusted value. In contrast, treatment of the LBF group at this concentration resulted in fewer differentially expressed genes (*n* = 60) (Figure 2B).

Clustered heatmaps (using Z score) were also generated for the first 50 most DGEs in *C. albicans* (HBF and LBF) samples treated with the 3.5 μg/mL of lactam compared to their positive controls (Figure 2C). Calculating Z‐scores helps to remove biases due to differences in sequencing depth or other technical factors between samples by identifying genes that are consistently up‐ or down‐regulated across samples, irrespective of the absolute expression levels [25]. A Z‐score of zero indicates that the gene's expression level is the same as the mean expression level across all samples, while a positive Z‐score indicates that the gene is expressed at a higher level than the mean, and a negative Z‐score indicates that the gene is expressed at a lower level than the mean. HBF treatment groups showed distinctly more upregulated genes (81 genes) in the lactam‐treatment samples compared to their untreated counterpart. The majority of these genes were related to plasma membrane transporters (*HGT14, DAL8*), cell wall protein (*CBK1*), exocytosis (*SEC1*), oxidative stress (*DAO2*) and vacuoles (C2_02490C_A). In contrast, the LBF treatment group displayed a gene expression profile that differed from that of the HBF group. However, most of the genes driving this difference lack functional annotation and did not contribute to any of the biological pathways enriched in the downstream analysis. Additionally, a list of the top 10 upregulated genes based on the log2 fold change in both treatment groups is shown in Table 2.

**TABLE 2:** List of the top 10 upregulated genes in HBF and LBF *C. albicans* treated with lactam at 4 h.

	Gene ID	Gene name	Log_2_ FC	*p* value (adj)
Up in HBF with 3.5 μg/mL	C2_02490C_A	C2_02490C_A	11.6260536	7.16E‐22
	C7_02320W_A	*SEC1*	8.09860661	5.73E‐38
	C3_05910W_A	*ZCF35*	7.64251078	1.23E‐05
	C1_10380C_A	*CBK1*	7.47551581	4.45E‐28
	C6_03180C_A	C6_03180C_A	7.24780403	7.01E‐05
	CR_07730W_A	*HGT14*	7.2122775	1.27E‐11
	C2_07060W_A	C2_07060W_A	6.99864618	2.00E‐09
	C4_06890W_A	*ARR3*	6.87187564	0.00075518
	CR_03750C_A	*PRP42*	6.85496237	0.00050894
	C1_11690W_A	C1_11690W_A	6.58828686	0.00029522
Up in LBF with 3.5 μg/mL	CR_09090C_A	CR_09090C_A	9.48691385	1.06E‐09
	CR_07730W_A	*HGT14*	9.08153434	4.78E‐07
	C7_02470C_A	C7_02470C_A	7.3361595	0.00013097
	CR_10780C_A	*IAH1*	6.90852133	0.0104844
	C3_02620C_A	C3_02620C_A	6.85771555	0.00129847
	C5_00700C_A	*SPE2*	6.83537683	0.00200544
	C7_00870W_A	C7_00870W_A	6.48632143	0.00269147
	CR_07750C_A	CR_07750C_A	5.64748829	0.04408891
	C7_02320W_A	*SEC1*	5.56356815	3.29E‐25
	C4_06780C_A	*OYE32*	5.53585189	0.03408796

Gene Ontology (GO) analysis of HBF treated with a higher concentration of lactam revealed a greater number of significantly enriched pathways (*p*adj ≤ 0.05) associated with downregulated genes (*n* = 19) compared with upregulated genes (*n* = 11). Pathways associated with downregulated genes were mainly related to biofilm formation and filamentous growth (Figure 2D). Hyphal growth‐related genes such as *HWP1, GCA2*, *ECE1* were significantly downregulated. Hyphal cell wall and cell adhesion pathways were also among the enriched biological functions related to downregulated genes. These findings suggest a pronounced antibiofilm effect of lactam, mainly by inhibiting fungal filamentation. A summary of the enriched biological processes among the downregulated genes in samples treated with lactam in both the HBF and LBF groups is presented in Figure S1.

On the other hand, genes involved in the transition metal ion binding pathway were among the most significantly upregulated in *Candida* cells treated with 3.5 μg/mL (*p*adj = 0.001). This pathway is involved in many cellular aspects within the *C. albicans* cell including metabolism, oxidative stress response, virulence, and adaptation to host environments. Also, pathways related to secondary active transmembrane transporter activity, cell wall, cell–cell adhesion, and zinc ion binding were among the significantly enriched pathways associated with upregulated genes in this treatment group (Figure S2).

For LBF, pathways related to response to cellular starvation, nutrient levels, and response to external stimulus were significantly downregulated. While the active ion transmembrane activity was among the enriched pathways related to upregulated genes in HBF group, this pathway was associated with both upregulated and downregulated genes in this treatment category (Figure 2E). An example of the key enriched pathways related to the upregulated genes in the treatment category is the organic hydroxy compound metabolic process pathway, which is responsible for the metabolism of carbohydrates and the antioxidant defence function through the pentose phosphate pathway. The protein dephosphorylation pathway was also among the enriched pathways in the LBF group treated with the higher concentration of lactam. Genes found to be associated with this pathway were *OCA1, OCA6, PTP3*. All these genes are considered protein tyrosine phosphatases and play a major role in stress response such as oxidative stress, and also regulate the yeast to hyphal morphogenesis (Figure S3).

### 3.3 Functional Analysis of RNA‐Seq Findings

**FIGURE 3:**
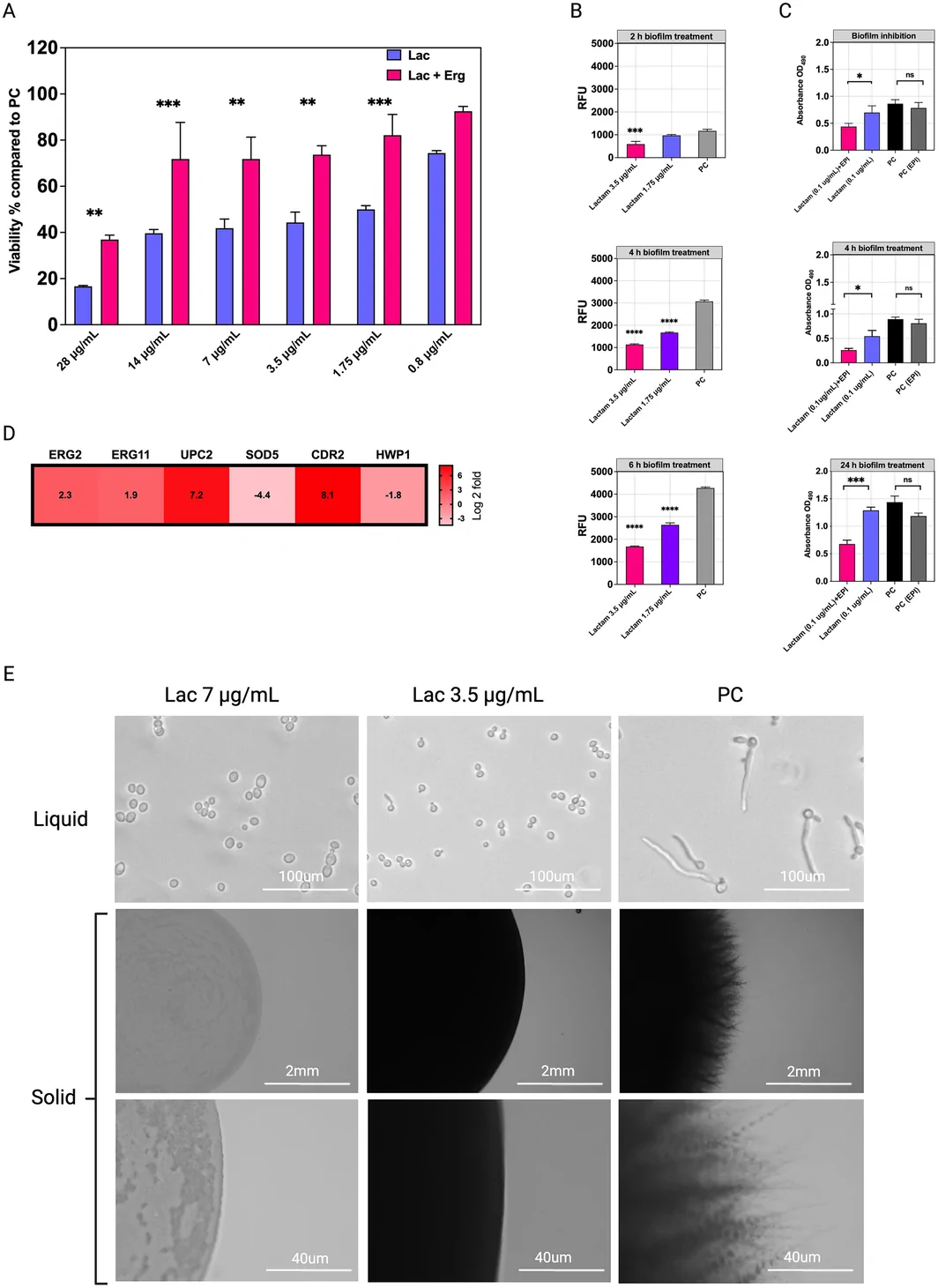
Mechanistic analysis to assess the mode of action of lactam. (A) Addition of exogenous ergosterol restores viability and growth of *C. albicans* cells subjected to lactam treatment. Different concentrations of lactam were tested in the presence or absence of exogenous ergosterol (50 μM) for their biofilm inhibiting ability after 4 h of incubation. XTT viability assay was utilised for assessment. (B) *C. albicans* biofilms display a dose‐dependent efflux activity in the presence of lactam. 24 h of *C. albicans* biofilms were incubated with Ala‐Nap (128 mg/L) for 2 h, 4 h, and 6 h, and resultant fluorescent production was measured using plate reader. High values indicate low efflux and vice versa. *C. albicans* biofilms. (C) Sensitivity of *C. albicans* cells and biofilms to lactam has increased in the presence of an efflux pump inhibitor (EPI). A subinhibitory concentration of lactam (0.1 μg/mL) was tested against *C. albicans* cells and early (4 h) and late (24 h) biofilms in the presence and absence of EPI (64 μg/mL). Viability was assessed using XTT assay. PC refers to positive untreated control. (D) Heatmaps showing expression levels of some *C. albicans* key genes related to ergosterol biosynthesis, oxidative stress, drug efflux pumps, and hyphal morphogenesis in response to lactam. Expression of these genes were measured in *C. albicans* 4 h biofilm treated with lactam for 4 h. Data shown is the mean log2 fold change relative to untreated biofilms. Gene expression was assessed using real time qPCR and percentage expression was normalised to *ACT1* housekeeping gene. (E) The anti‐filamentation activity of lactam against cells of *C. albicans* in solid and liquid media. Two concentrations of lactam have been tested against *C. albicans* reference strain SC5314. For liquid assays, lactam‐treated and untreated cells were imaged 3 h after treatment, whereas for solid media, colonies were imaged 4–5 days after incubation. White arrows indicate filamentous structure of interested cells. Images were taken 4–5 days. PC refers to untreated biofilms, and RFU refers to relative fluorescence units. Statistical significance was presented as *p < 0.05, **p < 0.01, ***p < 0.001, ****p < 0.0001.

Sterol pathway and plasma cell membrane were one of the enriched pathways in LBF fungal cells in response to lactam treatment (Figure 2E). Additionally, multiple genes (*EBP1*, *UGT51C1*, *UPC2*) implicated in the ergosterol biosynthesis process were found to be highly upregulated in the HBF treatment group. Simultaneous addition of ergosterol and lactam to *C. albicans* cells restored cell growth as demonstrated by the XTT assay (Figure 3A), where 5 out of 6 concentrations of lactam tested showed a statistically significant increase in viability when ergosterol was added. These results overall suggest that the activity of lactam could be partly a result of ergosterol depletion.

Fungal efflux pumps are likely affected by lactam exposure, as suggested by transcriptional analysis, in which the active ion transmembrane transporter pathway was significantly enriched in both HBF and LBF treatment groups. Using an Ala‐Nap assay (quantifiable inferred efflux activity), lactam‐treated biofilms showed a time‐ and dose‐dependent increase in efflux pump activity at 2, 4, and 6 h compared to untreated biofilms (Figure 3B). This was indicated by low fluorescence emission in yeast cells treated with 3.5 μg/mL and 1.75 μg/mL of lactam, except at the 2‐h time point for the lower concentration (1.75 μg/mL). An efflux pump inhibitor (EPI) was subsequently used to evaluate the role of efflux pumps in the antifungal activity of lactam. The addition of EPI increased the sensitivity of *C. albicans* biofilms to a subinhibitory concentration of lactam, with a more pronounced effect observed in 24 h treated biofilms *p* < 0.001 compared to early biofilms of *C. albicans* *p* < 0.05 (Figure 3C).

Given the previously observed phenotypic changes, we next undertook a targeted gene expression analysis of the 4 h lactam‐treated biofilm. Ergosterol‐related genes, *ERG2* and *ERG11*, were significantly upregulated (Log_2_ FC = 2.3 and 1.9, respectively), along with *UPC2* (Log_2_ FC = 7.2), a transcription factor regulating ergosterol biosynthesis. *SOD5*, a major antioxidant gene, was among the most downregulated (Log_2_ FC = −4.4), suggesting reduction in oxidative stress tolerance in lactam‐treated early biofilms. *HWP1*, involved in hyphal morphogenesis, was also downregulated. In contrast, *CDR2*, a known drug resistance gene encoding efflux pumps, showed the highest upregulation (Log_2_ FC = 8.1) (Figure 3D).

To verify the role of lactam in inhibiting hyphal morphogenesis, a reference strain SC5314 *C. albicans* strain was treated with two subinhibitory concentrations of lactam in solid and liquid medium. Filamentation‐inducing and non‐inducing conditions were applied. Microscopic examination revealed that both lactam concentrations showed a remarkable inhibition of hyphal formation in both liquid and solid media when compared to untreated yeast cells (Figure 3E).

### 3.4 Lactam Could Have an Impact on Vacuole Function

**FIGURE 4:**
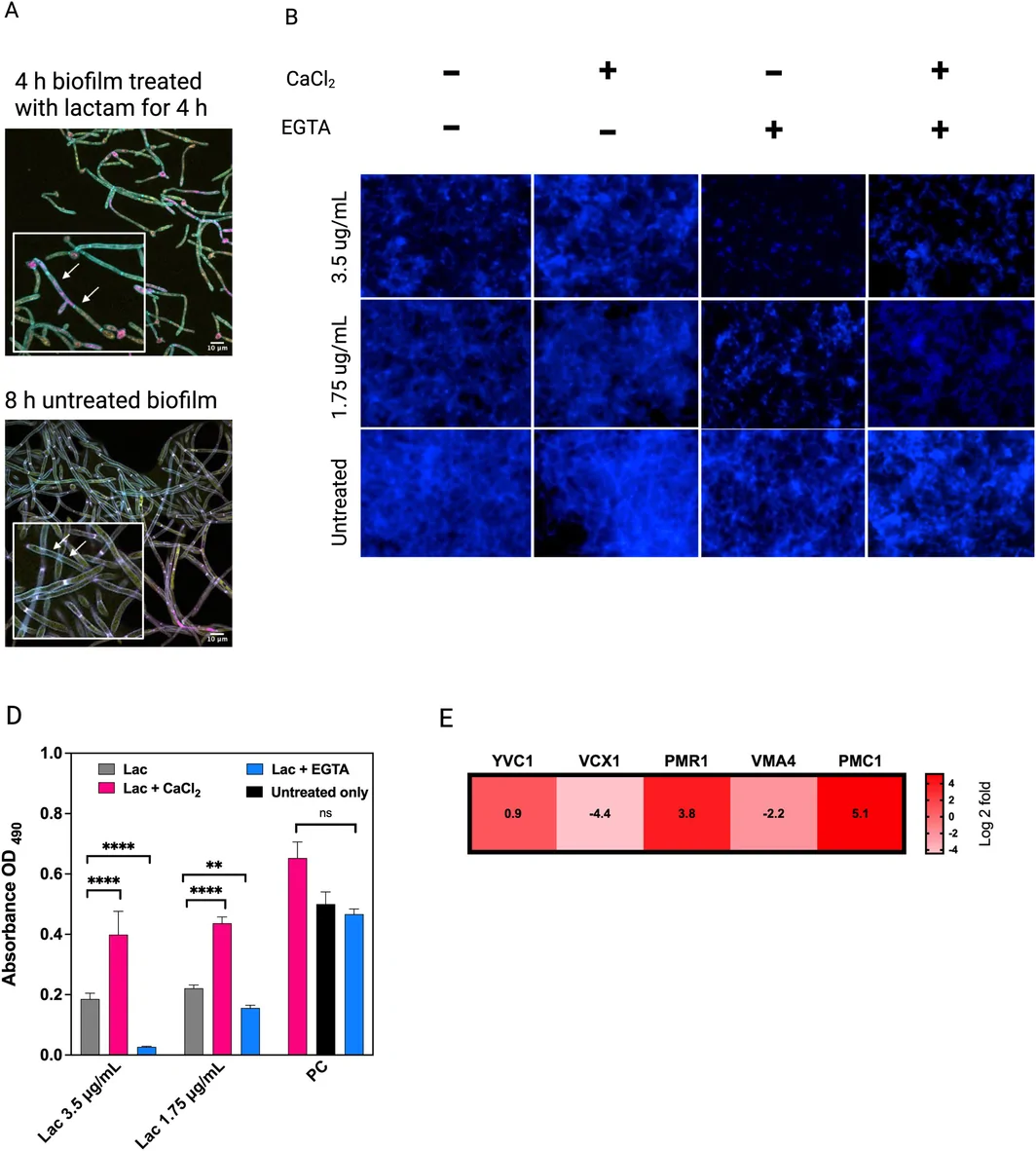
Investigating the effect of lactam on *Candida* vacuole function. (A) Confocal images showing 4 h biofilms of *C. albicans* (SC5314) treated with lactam (7 μg/mL) for 4 h and 8 h untreated control. Increased vacuole permeability was observed in lactam‐treated early biofilms of *C. albicans* indicated by increased fluorescence. Calcofluor white stained the fungal hyphae blue while 5‐(6)‐carboxy‐2′,7′‐dichlorofluorescein diacetate (C‐DCFDA) stained them green. Scale bar is 10 μm. White arrows indicate stained vacuoles' lumen in untreated biofilms and cytoplasm in lactam‐treated biofilms. (B) Fluorescence microscopic examination showing the effect of calcium on the antifungal activity of lactam. Upper two panels represent *C. albicans* cells treated with two concentrations of lactam in the presence or absence of CaCl_2_, EGTA, or their combinations. Untreated biofilms were also included. Calcofluor white was used to stain the fungal hyphae (blue). Scale bar is 100 μm at 40× magnification. (C) Lactam was tested in the presence or absence 10 mM of calcium chloride and a calcium chelator (EGTA). Viability was assessed using XTT assay. Data represents mean ± SD. Statistical significance between the treated and untreated cells was presented as **p < 0.01 and ****p < 0.0001. (D) Heatmaps showing expression levels of some *C. albicans* genes in response to Lactam. Expression levels of genes implicated in *C. albicans* calcium homeostasis and related to *Candida* vacuole. Data shown is the mean log2 fold change relative to untreated biofilms. Gene expression was assessed using real time qPCR and percentage expression was normalised to *ACT1* housekeeping gene.

Vacuolar membrane permeability (VMP) is a key indicator of oxidative stress‐induced cell death and vacuolar dysfunction in yeast [26]. C‐DCFDA is a useful VMP probe, as it could be hydrolysed to produce fluorescent FDA by acidic estrases which are normally localised in the vacuoles cavity but could be released to the cytosol when VMP is enhanced [27]. In this study, C‐DCFDA and CFW (for better visualisation) were used to stain treated and untreated *C. albicans* biofilms. Once inside the vacuole, C‐DCFDA is hydrolysed by intracellular esterases, producing a de‐esterified fluorescent product that is polar and therefore retained within the vacuolar lumen. A potential increase in vacuolar membrane permeability (VMP), resulting from elevated intracellular ROS in lactam‐treated fungal cells, was assessed using the fluorescent probe H_2_DCFDA (Figure 4A). Lactam‐treated biofilms exhibited markedly increased fluorescence relative to untreated controls, suggesting enhanced vacuolar membrane permeability following lactam exposure [28] (Figure 4A).

Since RNA‐seq data indicated that vacuole‐related genes were significantly differentially expressed (Table 2), and the VMP assay revealed differences between lactam‐treated and untreated biofilms, and given that the vacuole is a key site of Ca^2+^ storage, a simple and cost‐effective approach to assess the role of Ca^2+^ in lactam activity was to evaluate its antifungal effect in the presence and absence of an exogenous calcium source. Qualitative and quantitative assessments were performed, and fluorescence imaging revealed a substantial difference between lactam‐treated biofilms of *C. albicans* in the presence of CaCl_2_ and EGTA. Increased fluorescence was observed in fungal biofilms treated with two concentrations of lactam in the presence of a Ca^2+^ source compared to biofilms treated with lactam alone. In contrast, in the presence of EGTA, there was a marked reduction in fluorescence in treated biofilms compared to both Ca^2+^‐supplemented and lactam‐only treated biofilms. Untreated biofilms showed no significant difference in fluorescence intensity in the presence or absence of CaCl_2_ or EGTA (Figure 4B). XTT results showed that the addition of 10 mM of CaCl_2_ reduced the biofilm inhibiting activity of lactam (1.75, 3.5 μg/mL) by approximately two‐fold (*p* < 0.0001). Conversely, the addition of the Ca^2+^ chelator EGTA (10 mM) enhanced lactam activity significantly. Approximately 10‐fold increase (*p* < 0.0001) in the biofilm inhibiting ability was recognised with cells treated with 3.5 μg/mL of lactam and EGTA compared to lactam‐only treated cells. CaCl_2_ and EGTA at the concentrations tested had no visible effects on *C. albicans* growth when tested individually (Figure 4C).

Lactam treatment significantly altered the expression of all tested calcium‐related genes in *C. albicans* biofilms. The vacuolar calcium transporter genes *PMC1* and *VCX1* showed differential expression, with *PMC1* strongly up‐regulated (Log_2_ FC = 5.1) and *VCX1* significantly downregulated (Log_2_ FC = −4.4). The *YVC1* gene, involved in vacuolar calcium release, was moderately down‐regulated (Log_2_ FC = −0.9). *VMA4*, encoding a V‐ATPase subunit crucial for vacuolar acidification and cellular stress response, was significantly downregulated (Log_2_ FC = −2.2). PMR1, a Golgi calcium pump important for detoxifying cytosolic Ca^2+^, was significantly upregulated (Log_2_ FC = 3.8), suggesting disturbed calcium homeostasis in lactam‐treated cells (Figure 4D).

### 3.5 Combining Lactam With Antimicrobial Agents Showed Synergistic Effect

**FIGURE 5:**
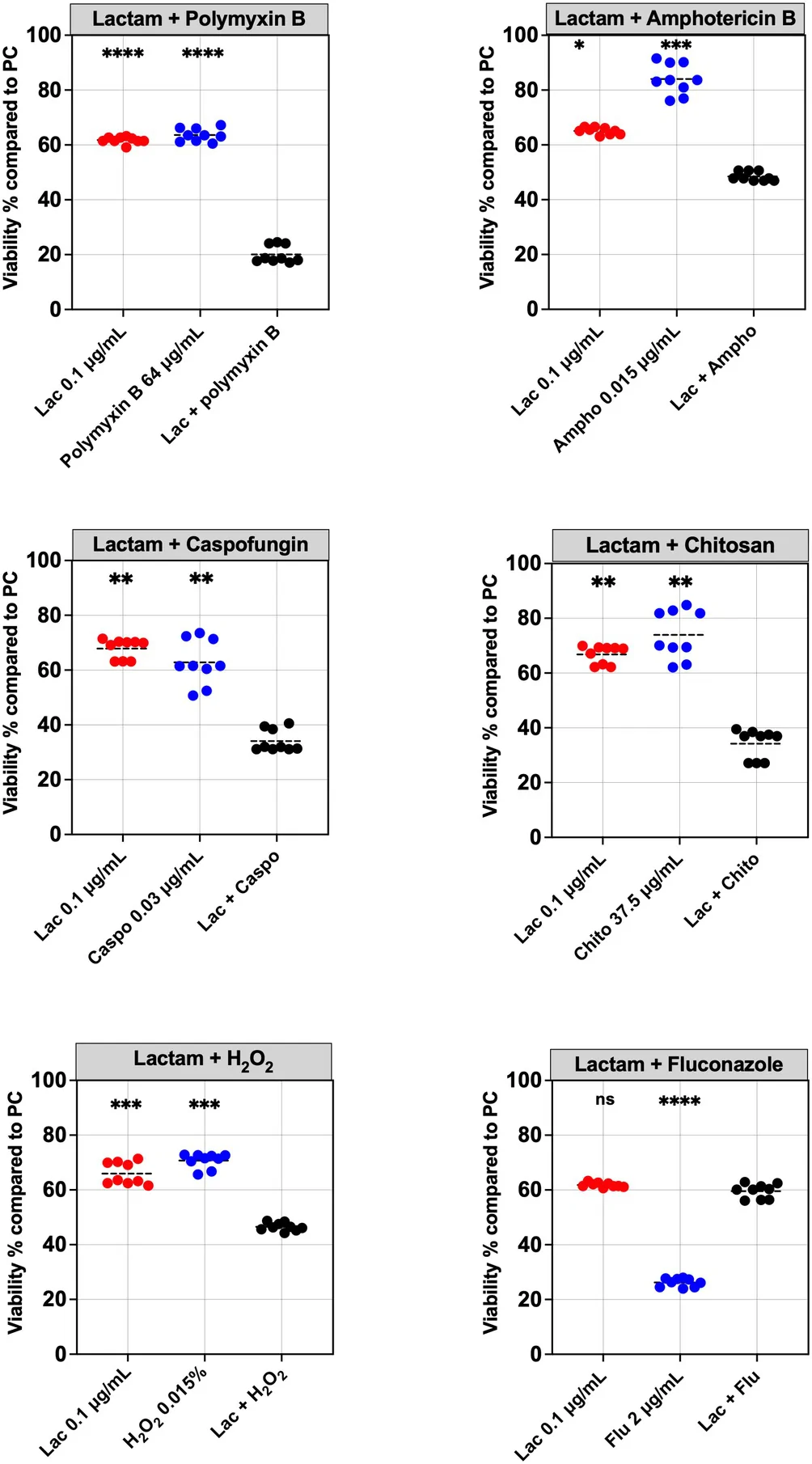
Combination treatment of lactam with a number of antimicrobial agents. The biofilm inhibiting activity of lactam ± polymyxin B, amphotericin B, caspofungin, chitosan, H_2_O_2_, and fluconazole. Viability was assessed using XTT assay, and percentage of inhibition was calculated relative to positive control. Statistical significance was presented as *p < 0.05, **p < 0.01, ***p < 0.001, ****p < 0.0001.

To further augment the biofilm inhibiting properties of lactam, several compounds that have either an antibacterial or antifungal effect were tested with lactam against *C. albicans* (SC5314) cells. A subinhibitory concentration of each drug was selected (based on data from the checkerboard assay) and tested against cells of *C. albicans* ± lactam. A significant synergistic antifungal effect was observed with all drug combinations, except those involving fluconazole. An antagonistic interaction was found when lactam was combined with fluconazole at the subinhibitory concentration tested (Figure 5). XTT results showed a significant reduction in fungal cell metabolic activity when lactam was combined with polymyxin B, resulting in 79.6% viability (*p* < 0.0001), compared to polymyxin B alone (36.2%) or lactam alone (37.8%). Similar synergistic effects were observed with amphotericin B, as combining 0.015 μg/mL of this antifungal compound with 0.1 μg/mL lactam produced significant inhibitory activity (*p* < 0.001). Additionally, chitosan, H_2_O_2_, and caspofungin each enhanced the antifungal effect of lactam, yielding viability percentages of 34.2% (*p* < 0.01), 46.5% (*p* < 0.01), and 34.1% (*p* < 0.001), respectively, compared to 67.8% in yeast cells treated with lactam alone.

## 4 Discussion

The antifungal discovery pipeline has for many years been neglected, though recently there has been a resurgence of activity [29] due to the prominence of fungal diseases on the global stage. The works described herein aim to contribute to the arsenal of available therapies to manage fungal diseases. Here, we describe how naturally derived lactam molecules have the capacity to inhibit and kill *C. albicans* . Through a combination of transcriptomics, biochemical and microscopical analyses, we demonstrate that the lactam molecule appears to gradually induce cytotoxic effects and interfere with subsequent hyphal growth and proliferation. Despite evidence of cellular protective responses, including upregulation of efflux systems and stress‐associated proteins, membrane integrity appears to be compromised, accompanied by increased vacuolation, which may represent a cellular response to mitigate toxicity. Collectively, these findings suggest that lactam may have potential as an antifungal agent for biofilm control across a range of applications.

Initially, the antifungal activity against *C. albicans* reference strain and LBF and HBF clinical isolates were examined by observing their growth kinetics. Lactam was shown to have a greater inhibitory activity against the HBF (hyphal‐dominant phenotype) than the LBF (yeast‐dominant phenotype). We have previously concluded that LBF *C. albicans* showed greater tolerance than HBF when treated with sodium hypochlorite [12]. Hyphal morphogenesis was shown to be exquisitely sensitive to the effects of lactam, as confirmed by the filamentation assay and transcriptional analyses, with the effect being rapid (within the first 4 h). These phenotypic effects may explain the differential sensitivity/tolerance of hyphal dominant strains. Our data hint at some broad‐spectrum equivalent mode of action to that of hypochlorite [12]. Recently it has been shown that *GAP6, HSP21* and *PRN1* are possible key genes that protect *C. albicans* cells against HOCl [30], are involved in metabolic processes, ammonia transport and unfolded proteins response pathways. Of notable interest is the clear observation of a vacuolation phenotype within hyphae, which has been reported as a potential protective response following antifungal exposure [31, 32]. We therefore aimed to understand what genes and pathways were involved in lactam sensitivity.

We conducted a large‐scale transcriptional analysis, and in doing so revealed a significant degree of transcriptional changes when treated with lactam. As predicted from phenotypic studies, DGE was more prominent in the HBF group, which was also observed in other studies [33]. That may explain the tolerability of the LBF strain to lactam where it is less affected at a molecular level. Conversely, DGE may simply be a logical reflection of the phenotypic changes opposed to lactam‐specific effects per se. However, the top 10 significantly up and downregulated genes were related to vacuole, oxidative stress, cell wall, and filamentous growth. This indicated that the analysis had picked up genes associated with more than the filamentation effects captured from phenotypic analysis, and also the importance of the vacuoles that rapidly formed our target hypothesis.

Increased vacuolar membrane permeability is commonly used as an indicator of vacuolar dysfunction [22] and was assessed here using a well‐established oxidant‐sensitive marker. The increased fluorescence observed in treated biofilms may reflect impaired vacuolar function in *Candida* cells in response to lactam exposure. Previous studies have reported that vacuolar dysfunction in *C. albicans* is associated with increased reactive oxygen species (ROS) levels, which can in turn affect Ca^2+^ transport from the vacuole to the cytosol [22]. Additionally, disruption of vacuolar function, particularly of the vacuolar ATPase, has also been shown to influence the activity of key vacuolar membrane transporters, including the Ca^2+^ pump Pmc1, the Ca^2+^/H^+^ exchanger Vcx1, and the Ca^2+^ channel Yvc1, all of which are involved in Ca^2+^ homeostasis [34].

Accordingly, the effect of lactam treatment on genes associated with cellular calcium homeostasis was examined. Lactam exposure was associated with altered expression of several key genes, including *PMC1*, *VCX1*, *YVC1*, and *PMR1*. Notably, *PMC1* was strongly upregulated (log_2_ fold change = 5.1). Upregulation of *PMC1* has previously been linked to activation of the calcineurin signalling pathway and increased tolerance to antifungal agents such as fluconazole [35, 36], which may partially account for the reduced sensitivity to lactam observed here. Furthermore, modulation of extracellular calcium levels in the presence of lactam significantly altered its antifungal activity, with calcium supplementation reducing, and calcium depletion enhancing, its efficacy. Similar effects have been reported for azole antifungals, where changes in calcium availability influence antifungal activity through regulation of calcium‐dependent signalling pathways [37]. However, further work is required to validate these findings. This may include the use of CDCFDA and FM4‐64, which have been successfully used to visualise vacuoles in *C. albicans* , with the dyes exhibiting distinct fluorescence spectra and localising to the vacuolar lumen and membrane, respectively [28]. Additionally, intracellular free Ca^2+^ levels can be further quantified using a Ca^2+^‐sensitive indicator such as Fura‐2 AM, enabling detection of changes in cytosolic Ca^2+^ [38].

Further downstream functional analysis was able to capture some insights to support the notion that *C. albicans* appears to undergo some form of toxicity to lactam treatment, which gains intracellular access and is likely to be concentrated into vacuoles. The cells respond to its effects through strengthening of the cell wall and efflux of the molecule as it enters the cell. Many of the responses appear to be related to tolerance, but the lactam eventually overcomes the cell, leading to cell death. Assessing the impact of lactam on hyphal morphogenesis was a major aspect of the functional analysis of transcriptomic data. Lactam‐treated *C. albicans* cells revealed a number of differentially expressed genes and several enriched pathways that were proven to negatively impact fungal filamentation. To further verify this phenotypic effect, a filamentation assay using hypha‐inducing conditions was performed. Microscopic examination revealed a clear inhibition of filamentation in *C. albicans* reference strain SC5314 both in liquid and solid media. This outcome can be due to several lactam‐related effects. For example, vacuole dysfunction, increased oxidative stress and imbalance in metal ions are all adverse outcomes that eventually inhibit hyphal morphogenesis in *C. albicans* cells when treated with lactam [39]. Transcriptional analysis of *HWP1*, a gene encoding a key protein involved in adhesion, biofilm formation, and hyphal morphogenesis [40], showed downregulation, implying a decrease in *C. albicans* ' hyphal morphogenesis ability. This is likely linked to the toxic effects elicited by the lactam rather than a specific inhibition of transcription factors driving the process per se, but further studies are required.

Ergosterol, a key component of fungal cell membranes absent in mammalian cells, is a valuable antifungal target [41]. While primarily located in the plasma membrane, ergosterol is also found in organelles like mitochondria and vacuoles [19]. In lactam‐treated *C. albicans* cells, the sterol biosynthesis pathway was enriched, prompting investigation into ergosterol's role. Supplementing ergosterol restored cell viability after 4 h of lactam exposure, and genes involved in ergosterol biosynthesis (*ERG2*, *ERG11*, and *UPC2*) were highly upregulated. Similar responses have been reported with fluconazole and amphotericin B treatments [42]. A possible mechanism is that vacuole membrane damage from lactam induces ergosterol trafficking from the plasma membrane to maintain membrane integrity. An alternative explanation is that lactam impairs cell membrane function in *C. albicans* , consistent with observations reported for other antifungal agents. Restoration of compromised membranes through supplementation with exogenous ergosterol has been shown to enhance tolerance to antifungal exposure [43]. Accordingly, quantification of cellular ergosterol content, alongside assessment of membrane permeability, represents a logical direction for future investigation.

Given these effects, we aimed to assess whether we could augment the effect of the lactam through combination therapy. Similar to lactam characteristics, polymyxin B was found to induce a vacuolar phenotypic change in *Aspergillus niger* [44], and therefore it was hypothesised that it will enhance the antifungal activity of lactam. Indeed, polymyxin B showed a significant synergistic relationship, suggesting shared characteristics. This enhanced antifungal effect was also observed when this antibiotic was combined with amphotericin B and fluconazole against different fungi [45]. Given that both the cell wall and membrane were also shown to be impacted by lactam, both caspofungin and chitosan were tested in combination with lactam as they are both known to impact the fungal cell wall. Both enhanced the biofilm‐inhibiting effects of lactam, likely due to their shared targeting of the *FKS1* gene. This is a key component of the fungal cell wall that was overexpressed in lactam‐treated samples. Amphotericin B, on the other hand, has been reported to possess a vacuole‐disrupting effect [46] which may explain the augmented antifungal effect when combining this antifungal with lactam. Similarly, combining lactam with H_2_O_2_, a well‐recognised oxidative stress‐inducing agent has synergised their biofilm inhibiting properties, supporting the findings of the differential expression of several oxidative‐stress related genes in yeast cells treated with lactam. Combination therapies provide several key benefits, such as lowering the risk of antimicrobial resistance, enhancing antimicrobial efficacy at lower doses, shortening treatment duration, and minimising drug‐related toxicities [47]. These studies also lend themselves to understanding the mode of action of novel compounds.

In conclusion, this study investigated the antifungal potential of a novel compound, lactam, using phenotypically distinct strains of *C. albicans* . The findings further confirmed the impact of lactam on various cellular processes in fungal cells, as demonstrated by transcriptomic and mechanistic analyses. However, it is well recognised that biofilms in both health and disease often have a polymicrobial nature and commonly include fungi. Therefore, investigating the antifungal activity of lactam in a more polymicrobial environment represents a logical next step.

## Funding

This work was supported by Unilever and National Biofilms Innovation Centre, 04POC21‐293.

## Ethics Statement

The authors have nothing to report.

## Consent

All authors read and approved this manuscript.

## Conflicts of Interest

The authors declare no conflicts of interest, except Joanne O'Keefe and Neil Parry who work for Unilever, and Yvonne Davies who works for Pehnros Bio.

## Supporting information


**Figure S1:** Enriched biological processes within the downregulated genes in samples treated with Lactam (3.5 μg/mL).
**Figure S2:** Enriched biological processes within the upregulated genes in HBF samples treated with Lactam (3.5 μg/mL).
**Figure S3:** Enriched biological processes within the upregulated genes in LBF samples treated with Lactam (3.5 μg/mL).

## Data Availability

The data that support the findings of this study are available from the corresponding author upon reasonable request.

## References

[1] G. Ramage , R. Kean , R. Rautemaa‐Richardson , C. Williams , and J. L. Lopez‐Ribot , “Fungal Biofilms in Human Health and Disease,” Nature Reviews. Microbiology 23 (2025): 355–370. 39910237 10.1038/s41579-025-01147-0

[2] WHO , “WHO Fungal Priority Pathogens List to Guide Research, Development and Public Health Action,” (2022).

[3] S. M. Noble , B. A. Gianetti , and J. N. Witchley , “ *Candida albicans* Cell‐Type Switching and Functional Plasticity in the Mammalian Host,” Nature Reviews. Microbiology 15 (2017): 96–108. 27867199 10.1038/nrmicro.2016.157 PMC5957277

[4] G. Ramage , R. Rajendran , L. Sherry , and C. Williams , “Fungal Biofilm Resistance,” International Journal of Microbiology 2012 (2012): 528521. 22518145 10.1155/2012/528521 PMC3299327

[5] O. A. Cornely , R. Sprute , M. Bassetti , et al., “Global Guideline for the Diagnosis and Management of Candidiasis: An Initiative of the ECMM in Cooperation With ISHAM and ASM,” Lancet Infectious Diseases 25 (2025): e280–e293. 39956121 10.1016/S1473-3099(24)00749-7

[6] G. Ramage , E. Borghi , C. F. Rodrigues , R. Kean , C. Williams , and J. Lopez‐Ribot , “Our Current Clinical Understanding of Candida Biofilms: Where Are We Two Decades on?,” APMIS 131 (2023): 636–653. 36932821 10.1111/apm.13310

[7] M. Hoenigl , A. Arastehfar , M. C. Arendrup , et al., “Novel Antifungals and Treatment Approaches to Tackle Resistance and Improve Outcomes of Invasive Fungal Disease,” Clinical Microbiology Reviews 37 (2024): e0007423. 38602408 10.1128/cmr.00074-23 PMC11237431

[8] H. Abduljalil , O. A. Alshanta , S. Chougule , et al., “Lactams Exhibit Potent Antifungal Activity Against Monospecies and Multispecies Interkingdom Biofilms on a Novel Hydrogel Skin Model,” APMIS 133 (2025): e13510. 39791268 10.1111/apm.13510 PMC11718591

[9] W. K. Goh , G. Iskander , D. S. Black , and N. Kumar , “An Efficient Lactamization of Fimbrolides to Novel 1, 5‐Dihydropyrrol‐2‐Ones,” Tetrahedron Letters 48 (2007): 2287–2290.

[10] S. Cui and E. Kim , “Quorum Sensing and Antibiotic Resistance in Polymicrobial Infections,” Communicative & Integrative Biology 17 (2024): 2415598. 39430726 10.1080/19420889.2024.2415598 PMC11487952

[11] B. J. Coco , J. Bagg , L. J. Cross , A. Jose , J. Cross , and G. Ramage , “Mixed Candida Albicans and Candida Glabrata Populations Associated With the Pathogenesis of Denture Stomatitis,” Oral Microbiology and Immunology 23 (2008): 377–383. 18793360 10.1111/j.1399-302X.2008.00439.x

[12] O. A. Alshanta , S. Shaban , C. J. Nile , W. McLean , and G. Ramage , “ *Candida albicans* Biofilm Heterogeneity and Tolerance of Clinical Isolates: Implications for Secondary Endodontic Infections,” Antibiotics (Basel) 8 (2019): 8. 31671533 10.3390/antibiotics8040204 PMC6963865

[13] G. Ramage , K. Vande Walle , B. L. Wickes , and J. L. Lopez‐Ribot , “Standardized Method for In Vitro Antifungal Susceptibility Testing of *Candida albicans* Biofilms,” Antimicrobial Agents and Chemotherapy 45 (2001): 2475–2479. 11502517 10.1128/AAC.45.9.2475-2479.2001 PMC90680

[14] B. D. Alexander , G. W. Procop , P. Dufresne , et al., “Reference Method for Broth Dilution Antifungal Susceptibility Testing of Yeasts,” (2002), In: CLSI document.

[15] E. McKloud , C. Delaney , L. Sherry , et al., “Recurrent Vulvovaginal Candidiasis: A Dynamic Interkingdom Biofilm Disease of Candida and Lactobacillus,” mSystems 6 (2021): e0062221. 34374560 10.1128/mSystems.00622-21 PMC8407231

[16] B. Short , C. Delaney , E. McKloud , et al., “Investigating the Transcriptome of *Candida albicans* in a Dual‐Species *Staphylococcus aureus* Biofilm Model,” Frontiers in Cellular and Infection Microbiology 11 (2021): 791523. 34888261 10.3389/fcimb.2021.791523 PMC8650683

[17] A. M. Bolger , M. Lohse , and B. Usadel , “Trimmomatic: A Flexible Trimmer for Illumina Sequence Data,” Bioinformatics 30 (2014): 2114–2120. 24695404 10.1093/bioinformatics/btu170 PMC4103590

[18] K. J. Livak and T. D. Schmittgen , “Analysis of Relative Gene Expression Data Using Real‐Time Quantitative PCR and the 2(‐Delta Delta C(T)) Method,” Methods 25 (2001): 402–408. 11846609 10.1006/meth.2001.1262

[19] Y. Q. Zhang , S. Gamarra , G. Garcia‐Effron , S. Park , D. S. Perlin , and R. Rao , “Requirement for Ergosterol in V‐ATPase Function Underlies Antifungal Activity of Azole Drugs,” PLoS Pathogens 6 (2010): e1000939. 20532216 10.1371/journal.ppat.1000939 PMC2880581

[20] R. Rajendran , E. Mowat , E. McCulloch , et al., “Azole Resistance of Aspergillus Fumigatus Biofilms Is Partly Associated With Efflux Pump Activity,” Antimicrobial Agents and Chemotherapy 55 (2011): 2092–2097. 21321135 10.1128/AAC.01189-10 PMC3088214

[21] J. Azadmanesh , A. M. Gowen , P. E. Creger , N. D. Schafer , and J. R. Blankenship , “Filamentation Involves Two Overlapping, but Distinct, Programs of Filamentation in the Pathogenic Fungus *Candida albicans* ,” G3: Genes, Genomes, Genetics 7 (2017): 3797–3808. 28951491 10.1534/g3.117.300224 PMC5677161

[22] Q. Yu , B. Zhang , B. Yang , et al., “Interaction Among the Vacuole, the Mitochondria, and the Oxidative Stress Response Is Governed by the Transient Receptor Potential Channel in *Candida albicans* ,” Free Radical Biology & Medicine 77 (2014): 152–167. 25308698 10.1016/j.freeradbiomed.2014.09.011

[23] L. E. Bouillet , A. S. Cardoso , E. Perovano , et al., “The Involvement of Calcium Carriers and of the Vacuole in the Glucose‐Induced Calcium Signaling and Activation of the Plasma Membrane H(+)‐ATPase in *Saccharomyces cerevisiae* Cells,” Cell Calcium 51 (2012): 72–81. 22153127 10.1016/j.ceca.2011.10.008

[24] H. Abduljalil , A. Bakri , K. Albashaireh , et al., “Screening the Tocriscreen Bioactive Compound Library in Search for Inhibitors of Candida Biofilm Formation,” APMIS 130 (2022): 568–577. 35791082 10.1111/apm.13260 PMC9541805

[25] Y. Zhao , M. C. Li , M. M. Konate , et al., “TPM, FPKM, or Normalized Counts? A Comparative Study of Quantification Measures for the Analysis of RNA‐Seq Data From the NCI Patient‐Derived Models Repository,” Journal of Translational Medicine 19 (2021): 269. 34158060 10.1186/s12967-021-02936-w PMC8220791

[26] H. Kim , A. Kim , and K. W. Cunningham , “Vacuolar H+‐ATPase (V‐ATPase) Promotes Vacuolar Membrane Permeabilization and Nonapoptotic Death in Stressed Yeast,” Journal of Biological Chemistry 287 (2012): 19029–19039. 22511765 10.1074/jbc.M112.363390 PMC3365936

[27] H. H. Agus , C. Sarp , and M. Cemiloglu , “Oxidative Stress and Mitochondrial Impairment Mediated Apoptotic Cell Death Induced by Terpinolene in Schizosaccharomyces Pombe,” Toxicology Research (Cambridge) 7 (2018): 848–858. 10.1039/c8tx00100f PMC6116180 30310662

[28] A. Richards , N. A. Gow , and V. Veses , “Identification of Vacuole Defects in Fungi,” Journal of Microbiological Methods 91 (2012): 155–163. 22902527 10.1016/j.mimet.2012.08.002

[29] M. Hoenigl , R. Sprute , M. Egger , et al., “The Antifungal Pipeline: Fosmanogepix, Ibrexafungerp, Olorofim, Opelconazole, and Rezafungin,” Drugs 81 (2021): 1703–1729. 34626339 10.1007/s40265-021-01611-0 PMC8501344

[30] C. S. Krishnan , T. J. Milne , G. R. Tompkins , R. D. Cannon , and E. Lamping , “Molecular Mechanism of Action of HOCl From Neutral‐pH Electrolysed Oxidising Water Against *Candida albicans* ,” Journal of Fungi 11 (2025): 761. 41295142 10.3390/jof11110761 PMC12653471

[31] H. Tournu , J. Carroll , B. Latimer , et al., “Identification of Small Molecules That Disrupt Vacuolar Function in the Pathogen *Candida albicans* ,” PLoS One 12 (2017): e0171145. 28151949 10.1371/journal.pone.0171145 PMC5289544

[32] N. Yamada , W. Murata , Y. Yamaguchi , K. I. Fujita , A. Ogita , and T. Tanaka , “Enhancing the Fungicidal Activity of Amphotericin B via Vacuole Disruption by Benzyl Isothiocyanate, a Cruciferous Plant Constituent,” Letters in Applied Microbiology 72 (2021): 390–398. 33128810 10.1111/lam.13425

[33] H. T. Taff , K. F. Mitchell , J. A. Edward , and D. R. Andes , “Mechanisms of Candida Biofilm Drug Resistance,” Future Microbiology 8 (2013): 1325–1337. 24059922 10.2217/fmb.13.101 PMC3859465

[34] L. Peng , Q. Yu , H. Zhu , et al., “The V‐ATPase Regulates Localization of the TRP ca(2+) Channel Yvc1 in Response to Oxidative Stress in *Candida albicans* ,” International Journal of Medical Microbiology 310 (2020): 151466. 33291030 10.1016/j.ijmm.2020.151466

[35] M. Karababa , E. Valentino , G. Pardini , A. T. Coste , J. Bille , and D. Sanglard , “CRZ1, a Target of the Calcineurin Pathway in *Candida albicans* ,” Molecular Microbiology 59 (2006): 1429–1451. 16468987 10.1111/j.1365-2958.2005.05037.x

[36] D. Sanglard , F. Ischer , O. Marchetti , J. Entenza , and J. Bille , “Calcineurin A of *Candida albicans* : Involvement in Antifungal Tolerance, Cell Morphogenesis and Virulence,” Molecular Microbiology 48 (2003): 959–976. 12753189 10.1046/j.1365-2958.2003.03495.x

[37] F. F. Liu , L. Pu , Q. Q. Zheng , et al., “Calcium Signaling Mediates Antifungal Activity of Triazole Drugs in the Aspergilli,” Fungal Genetics and Biology 81 (2015): 182–190. 25554700 10.1016/j.fgb.2014.12.005

[38] L. Sun and K. Liao , “The Effect of Honokiol on Ergosterol Biosynthesis and Vacuole Function in *Candida albicans* ,” Journal of Microbiology and Biotechnology 30 (2020): 1835–1842. 33263334 10.4014/jmb.2008.08019 PMC9728367

[39] C. Jia , Q. Yu , N. Xu , et al., “Role of TFP1 in Vacuolar Acidification, Oxidative Stress and Filamentous Development in *Candida albicans* ,” Fungal Genetics and Biology 71 (2014): 58–67. 25220074 10.1016/j.fgb.2014.08.012

[40] Y. Fan , H. He , Y. Dong , and H. Pan , “Hyphae‐Specific Genes HGC1, ALS3, HWP1, and ECE1 and Relevant Signaling Pathways in *Candida albicans* ,” Mycopathologia 176 (2013): 329–335. 24002103 10.1007/s11046-013-9684-6

[41] A. T. Sangamwar , U. D. Deshpande , and S. S. Pekamwar , “Antifungals: Need to Search for a New Molecular Target,” Indian Journal of Pharmaceutical Sciences 70 (2008): 423–430. 20046765 10.4103/0250-474X.44588 PMC2792545

[42] S. Schubert , K. S. Barker , S. Znaidi , et al., “Regulation of Efflux Pump Expression and Drug Resistance by the Transcription Factors Mrr1, Upc2, and Cap1 in *Candida albicans* ,” Antimicrobial Agents and Chemotherapy 55 (2011): 2212–2223. 21402859 10.1128/AAC.01343-10 PMC3088179

[43] M. Kodedova , M. Valachovic , and H. Sychrova , “The Replacement of Ergosterol With Alternative Sterols Affects the Physiological Function of the Yeast Plasma Membrane, Including Its H(+)‐ATPase Activity and Resistance to Antifungal Drugs,” Microbes and Infection 27 (2025): 105409. 39187062 10.1016/j.micinf.2024.105409

[44] A. Ogita , Y. Konishi , B. Borjihan , K. Fujita , and T. Tanaka , “Synergistic Fungicidal Activities of Polymyxin B and Ionophores, and Their Dependence on Direct Disruptive Action of Polymyxin B on Fungal Vacuole,” Journal of Antibiotics (Tokyo) 62 (2009): 81–87. 10.1038/ja.2008.13 19132057

[45] T. Pozzebon Venturini , L. Rossato , F. Chassot , et al., “In Vitro Synergistic Combinations of Pentamidine, Polymyxin B, Tigecycline and Tobramycin With Antifungal Agents Against Fusarium spp,” Journal of Medical Microbiology 65 (2016): 770–774. 27357655 10.1099/jmm.0.000301

[46] C. K. Kang , K. Yamada , Y. Usuki , A. Ogita , K. I. Fujita , and T. Tanaka , “Visualization Analysis of the Vacuole‐Targeting Fungicidal Activity of Amphotericin B Against the Parent Strain and an Ergosterol‐Less Mutant of *Saccharomyces cerevisiae* ,” Microbiology (Reading) 159 (2013): 939–947. 23475946 10.1099/mic.0.065714-0

[47] P. Zhu , Y. Li , T. Guo , et al., “New Antifungal Strategies: Drug Combination and Co‐Delivery,” Advanced Drug Delivery Reviews 198 (2023): 114874. 37211279 10.1016/j.addr.2023.114874

